# Sources of pain in laparoendoscopic gynecological surgeons: An analysis of ergonomic factors and proposal of an aid to improve comfort

**DOI:** 10.1371/journal.pone.0184400

**Published:** 2017-09-14

**Authors:** Sa Ra Lee, Sunah Shim, Taeri Yu, Kyungah Jeong, Hye Won Chung

**Affiliations:** Department of Obstetrics and Gynecology, College of Medicine, Ewha Womans University, Seoul, South Korea; Seoul National University Bundang Hospital, REPUBLIC OF KOREA

## Abstract

Minimally invasive surgery (MIS) offers cosmetic benefits to patients; however, surgeons often experience pain during MIS. We administered an ergonomic questionnaire to 176 Korean laparoscopic gynecological surgeons to determine potential sources of pain during surgery. Logistic regression analysis was used to identify factors that had a significant impact on gynecological surgeons’ pain. Operating table height at the beginning of surgery and during the operation were significantly associated with neck and shoulder discomfort (*P* <0.001). The ability to control the operating table height was the single factor most significantly associated with neck (*P* <0.001) and shoulder discomfort (*P* <0.001). Discomfort of the hand/digits was significantly associated with the trocar site (*P* = 0.035). The type of electrocautery activation switch and foot pedal were significantly related to surgeons’ foot and leg discomfort (*P* <0.001). In evaluating the co-occurrence of pain in 4 different sites (neck, shoulder, back, hand/digits), the neck and shoulder were determined to have the highest co-occurrence of pain (Spearman’s *ρ* = 0.64, *P* <0.001). These results provide guidance for identifying ergonomic solutions to reduce gynecological laparoscopic surgeons’ pain. Based on our results, we propose the use of an ergonomic surgical step stool to reduce physical pain related to performing laparoscopic operations.

## Introduction

Minimally invasive surgery (MIS) now accounts for the majority of surgical procedures due to its many advantages for patients [1]. However, an increasing number of laparoendoscopic surgeons (LES) suffer from musculoskeletal discomfort or pain. A recent study revealed that 86.9% of laparoscopic general surgeons suffer from physical discomfort [2,3]. Similarly, 88% of laparoscopic gynecological surgeons experienced physical discomfort, especially neck, shoulder, and back pain related to MIS [4]. These reports indicate the severity of this ergonomic problem and the urgent need to identify sources of pain and offer potential solutions to minimize the pain of surgeons performing MIS. Recently, the increasing number of endoscopic surgeries in various department emphasized the importance of awareness of ergonomics among not only the laparoscopic surgeon but also urologist and endoscopic sinus surgeon [5,6]. The maintenance in a prolonged static posture with flexion and extension of neck, shoulder, and upper extremities are reported to the main risk factors for injury for minimally invasive urologists [5].

Most such pain was reported to result from inappropriate positioning of endoscopic equipment or the surgeon’s poor stature [7]. Poor visualization and physical discomfort among the medical team were related to the type of equipment used for MIS [8]. In testing the task performance and neck muscle strain during laparoscopic suturing, the EMG activity was significantly affected by the monitor position during laparoscopic surgery [9]. Therefore, to decrease the discomfort of LES, it is important to identify anti-ergonomic factors in equipment that can cause pain or discomfort. The awareness and follow the guideline for ergonomics is also important. Wauben *et al*. reported pain distributions of LES; however, the heterogeneity of surgeons and of the surgical equipment in different departments, such as general surgery, urology, and gynecology, from different countries was a limitation of this study [10]. A study within a homogeneous LES group could be more meaningful; however, to our knowledge, no such studies have been reported to date. Therefore, the aim of this study was to determine the anti-ergonomic factors affecting laparoendoscopic gynecological surgeons (LEGS) and to propose a device that may aid in reducing discomfort arising from the identified ergonomic factors in a relatively homogeneous group of surgeons.

## Material and methods

### Materials

**Fig 1:**
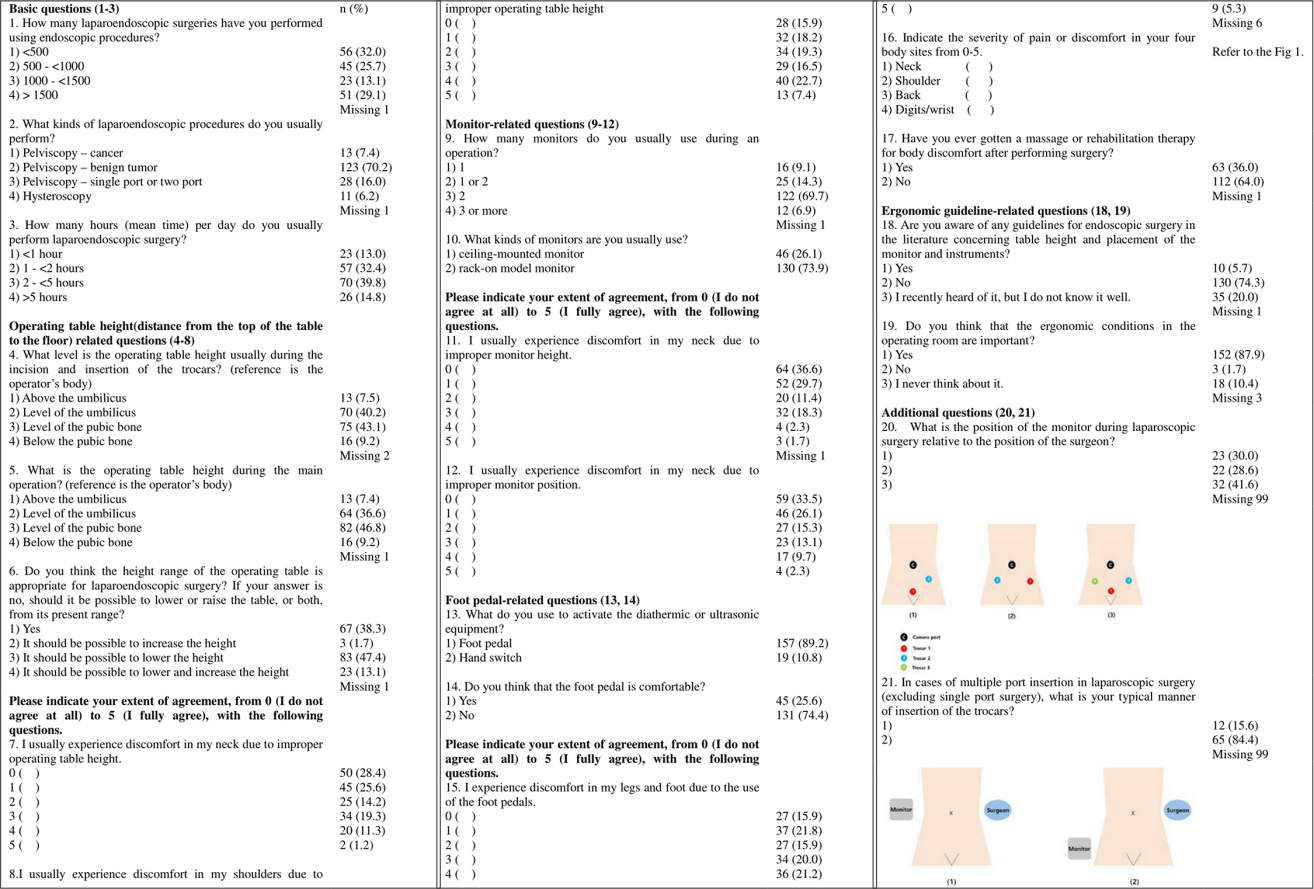
Questionnaire regarding sources of body pain in laparoendoscopic surgery.

We conducted a survey in relatively homogenous group of Korean LEGS and analyzed factors related to surgeons’ musculoskeletal pain. We used the summarized version of the questionnaire reported by Wauben et al, which consisted of 23 questions [8] and modified the questionnaire by selecting some original questions and adding others about the trocar site design and the relative position of the surgeon with respect to the position of the monitor (Fig 1). The questionnaire consisted of 21 questions regarding various aspects of endoscopic surgery, such as the type of monitor, operating table, endoscopic equipment, trocar site, and surgeons’ pain. Among 21 questions in this study, we adapted 17 questions (question no. 2–16, 18, 19) from the article by Wauben et al. and we added 4 questions (question no. 1, 17, 20, 21) for more investigation about ergonomics of LES.

A total of 398 emails were sent to Korean LEGS who are members of the Korean Society of Minimally Invasive Gynecology and board-certified obstetricians and gynecologists. This study was approved by the EWHA Womans University MokDong Hospital institutional review board (ECT 11-52-57).

### Statistical analysis

Response data in S1 File were analyzed using logistic regression analysis. The degrees of discomfort reported in different body parts were defined as response variables, and the other related features were defined as predictive variables. Because all of the response variables were ordinal data, we used the polr function in the MASS R statistical package, which implements a proportional odds assumption, to perform ordinal logistic regression analysis. After constructing a regression model, we performed an analysis of variance (ANOVA) to examine the significance of the model and each of the variables. In this case, we first constructed two regression models, with the variables either being tested or not. Next, we tested the significance of the differences between the models using ANOVA. All other statistical tests were also performed using R [11]. To identify the co-occurrence of pain severity between the four body sites assessed, we computed pair-wise Spearman rank correlation coefficients.

## Results

In total, 176 of 398 LEGS (52.4%) replied to the email questionnaire (number 1–19) with consent of participation to this study. We sent an additional 2 questions (number 20 and 21) regarding the monitor position and trocar type after the first round of questions, and the response rate to these questions was substantially lower. Only 40% (n = 79) of total participants replied to the additional questions. The mean age of respondents was 41.90 ± 6.46 years, and their mean height was 171.88 ± 6.42 cm. The majority of respondents (67.6%) were highly skilled, high output LEGS who had performed more than 500 laparoendoscopic surgeries. Most of the respondents (85.2%, n = 150) typically spent less than 5 hours per day performing laparoendoscopic surgery as an operator. Two-thirds (69.9%) of operations were for benign tumors, and 7% were for cancer. Fig 1 summarizes the responses to the questions of the questionnaire.

In the regression analysis of the relationship between pain in different body parts and factors described in the questionnaire, we adjusted age and height of LES. The main findings are as follows. First, operating table height-related variables including the table height at the beginning of operation and during the operation and the ability to control the table height were all significantly related to neck and shoulder discomfort. However, the degree of relation was stronger for shoulder discomfort (*P* <0.001) than for neck discomfort (*P <* 0.001) (Table 1). Accordingly, satisfaction with controlling the operating table height was significantly related to neck comfort. The shoulder discomfort was negatively correlated with age (*P* = 0.018).

**Table 1:** Correlation of operating table-related variables with neck and shoulder discomfort.

	Neck discomfort			Shoulder discomfort		
	*Coeff*	*SE*	*P value*	*Coeff*	*SE*	*P value*
**Age**	0.004	0.022	0.884	-0.055	0.023	**0.018**
**Height**	0.003	0.022	0.875	-0.025	0.023	0.290
**table.init.ht**	-0.111	0.243	0.650	-0.133	0.253	0.598
**table.op.ht**	-0.057	0.242	0.816	0.208	0.263	0.426
**ht.control2**	2.225	0.940	**1.011×10**^**−3**^	1.302	0.943	**2.012×10**^**−11**^
**ht.control3**	1.169	0.321		2.214	0.347	
**ht.control4**	1.611	0.441		2.490	0.470	

*Coeff*: regression coefficient, *SE*; standard error of regression coefficients, *P* value; ANOVA *P* value, table.init.ht; initial table height at the beginning of operation, table.op.ht; table height during the operation, ht.control; degree of height control during the operation. Note that the ht.control variable is categorical, with numbers 2, 3 and 4 indicating each category of the ht.control variable.

Second, in terms of monitor-associated variables, the ability to control the monitor height was the only variable significantly associated with neck discomfort in the ANOVA (*P* = 0.035), after adjusting age and height of LES. All other monitor-associated variables, including the number, type, and position of monitors relative to the position of the operator, were not significantly related to neck discomfort (Table 2).

**Table 2:** Correlation of monitor-related variables with neck discomfort.

	Neck discomfort–monitor height			Neck discomfort–monitor position		
	*Coeff*	*SE*	*P value*	*Coeff*	*SE*	*P value*
**Age**	-0.005	0.022	0.810	-0.042	0.022	0.051
**Height**	0.002	0.021	0.936	0.006	0.021	0.764
**Monitor number**	-0.011	0.208	0.959	-0.378	0.215	0.078
**Monitor type**	0.685	0.328	**0.036**	0.617	0.322	0.054

*Coeff*: regression coefficient, *SE*; standard error of regression coefficients, *P* value; ANOVA *P* value

**Fig 2:**
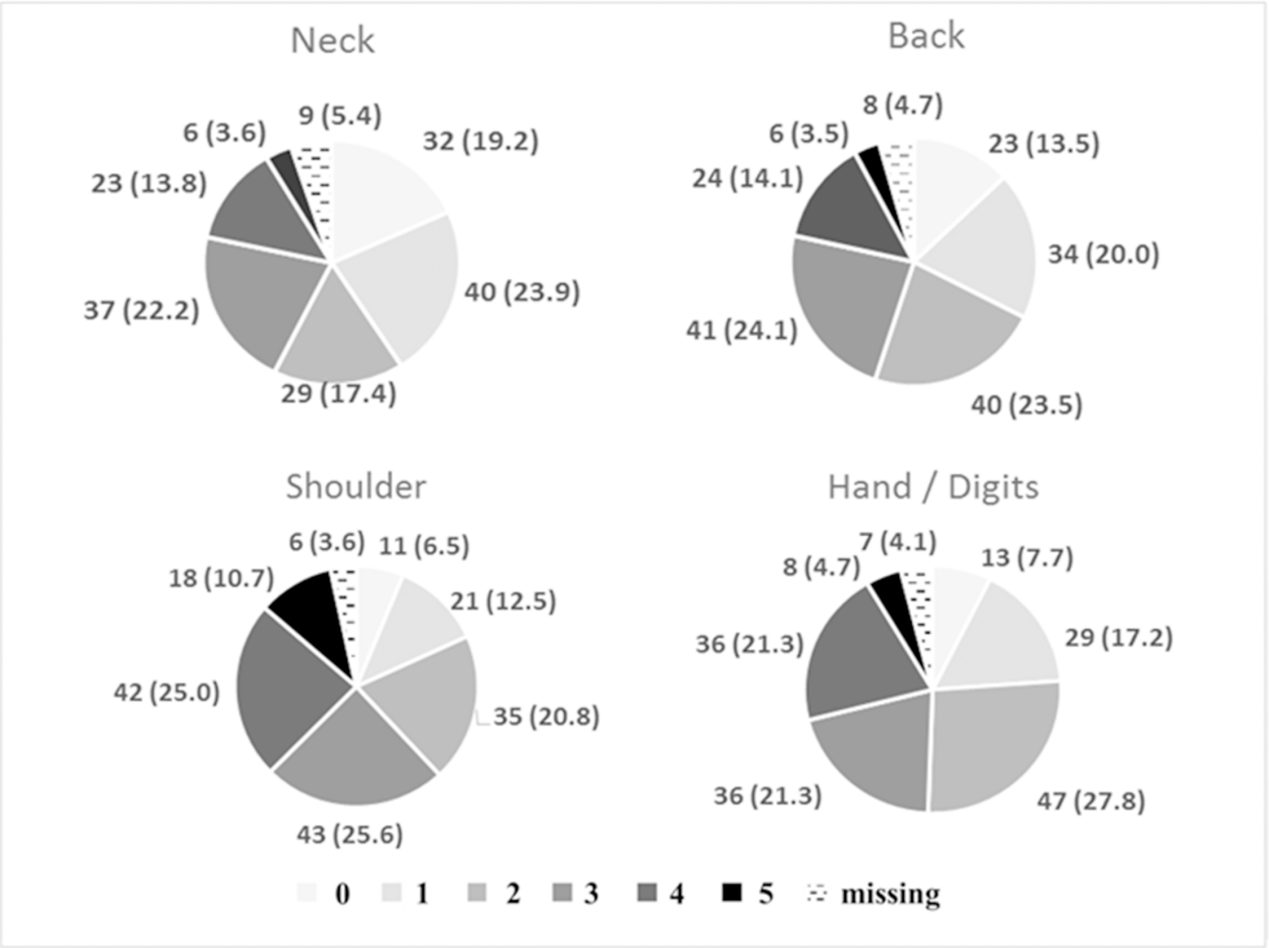
Distribution and severity of discomfort in four sites (answers to the question 16).

Third, in the questionnaire regarding degree of pain, to get information concerning pain sites and degrees of pain, the distribution and degrees of pain in four body parts (neck, shoulder, back, hand/digits) are summarized in Fig 2. Among pairs of locations, the two most significant correlations were between the severity of neck and shoulder discomfort (Spearman’s ρ = 0.64, *P* < 0.001) and between the severity of neck and back discomfort (Spearman’s *ρ* = 0.49, *P* < 0.001) (Table 3).

**Table 3:** Correlation of four sites of pain with each other.

	Neck	Shoulder	Back
**Neck**			
**Shoulder**	0.624		
**Back**	0.467	0.355	
**Hand/Digits**	0.283	0.376	0.343

Ordinal logistic regression analysis identified no significant relationships among the four pain sites, monitor position, and trocar sites, except for a significant association of hand/digits discomfort with trocar site (P = 0.035, Table 4).

**Table 4:** Correlation of pain in four sites with monitor position and trocar site design.

		Monitor position	Trocar site 2	Trocar site 3
**Neck discomfort**	*Coeff*	-1.071	0.078	0.509
	*SE*	0.619	0.625	0.586
	*P value*	0.083	0.556	
**Shoulder discomfort**	*Coeff*	-1.114	-0.374	0.166
	*SE*	0.660	0.592	0.551
	*P value*	0.089	0.543	
**Back discomfort**	*Coeff*	-1.061	0.452	0.417
	*SE*	0.610	0.609	0.551
	*P value*	0.080	0.706	
**Hand/Digits discomfort**	*Coeff*	-0.659	-0.626	0.689
	*SE*	0.582	0.599	0.546
	*P value*	0.258	**0.035**	

*Coeff*: regression coefficient, *SE*; standard error of regression coefficients, *P* value; ANOVA *P* value, Since the monitor position and trocar site were categorical variables, the numbers follow the variable names indicate different categories of the variables.

Finally, in the analysis of foot pedal-related variables, the feeling of comfort with the foot pedal and type of activation switch, foot pedal or hand switch, were related to pain in the foot or leg (Table 5). Foot or leg pain was strongly associated with surgeons’ reported comfort with the foot pedal and type of activation switch (*P* < 0.001). In the single-variable analysis, feeling comfortable with the foot pedal exhibited strong significance (*P* < 0.001) demonstrating a highly significant inverse association with the presence of foot pain, however the type of activation switch exhibited no statistical significance (*P* = 0.468).

**Table 5:** Correlation of discomfort in the foot or leg with feeling comfort with the foot pedal and type of foot pedal activation switch.

	Foot pedal comfort			Activation switch type		
	*Coeff*	*SE*	*P value*	*Coeff*	*SE*	*P value*
**Discomfort of foot or leg**	2.719	0.386	**1.765×10**^**−14**^	-0.350	0.483	0.468

Foot pedal comfort; feeling comfort with the foot pedal. Activation switch type; type of foot pedal activation switch. The two variables are categorical data. *Coeff*: regression coefficient, *SE*; standard error of regression coefficients, *P* value; ANOVA *P* value.

## Discussion

In this study, we demonstrated a significant relationship between endoscopic surgeons’ pain and their use of endoscopic instruments, which can provide important insight for the development of ergonomic solutions to address this pain.

We found that neck and shoulder discomfort were closely related to adjustability of the operation table height. Operating table height has been hypothesized to cause neck pain to surgeons performing exploratory laparotomy [12]. In general, LES tend to maintain a more static and extended neck posture than surgeons who perform laparotomy [13,14]. This finding indicates that the operating table height should be adjusted to the surgeon’s height, regardless of whether laparotomy or laparoendoscopy is being performed.

Optimizing the operating table height has also been previously reported to reduce surgeons’ shoulder pain [15]. Berquer et al. proposed that the optimal operating table height left laparoscopic instrument handles near the level of the surgeon’s elbow, based on not only subjective discomfort ratings but also deltoid and trapezius muscle electromyography (EMG) [16]. While the previous research focused on a fixed table height, we observed that the adjustability of operating table height was more strongly related to shoulder discomfort, as well as neck discomfort. These findings appear to be worth validating in future studies using equipment to measure muscle fatigue, such as EMG.

Interestingly, we found that the use of tower-mounted monitors was significantly related to the development of neck discomfort compared to the use of ceiling-mounted models, with an odds ratio of 1.98. This result suggests that the adjustability of monitor height might be helpful to relieve neck pain in LES.

There was marginal significance between neck pain and number of monitors used. This finding is not surprising, considering that as more monitors are used, the chance of overuse or rotating the neck decreases. Although this finding did not reach statistical significance in our study, the association was reported in previous report [17], therefore this association should be evaluate in the future studies.

The relationships among the four evaluated body sites reflect their anatomical proximity. The strongest correlation was between neck and shoulder pain, and the lowest was between neck and hand/digits pain. Monitor position and trocar site were not significantly related with the degree pain in the four sites. However, monitor position demonstrated a marginally significant relationship with neck, shoulder and back discomfort (*P* = 0.08, 0.09, and 0.08, respectively). These findings suggest that the adjustment of monitor position might be related to multiple sites of physical discomfort in LES.

Foot or leg pain was highly associated with foot-pedal comfort variables. This finding implies that even if surgeons use a foot pedal, the fit of the foot pedal determines surgeons’ comfort regardless of activation switch type.

The finding of most Korean gynecologic LES (74.3% of respondents) never heard about the ergonomic guideline for laparoscopic surgery remind us the importance of informing ergonomics. The ergonomics principles can benefit not only the LES in terms of fatigue, physical discomfort, and task efficiency, but also the patients who are undergoing the laparoscopic surgery for the same reasons.

**Fig 3:**
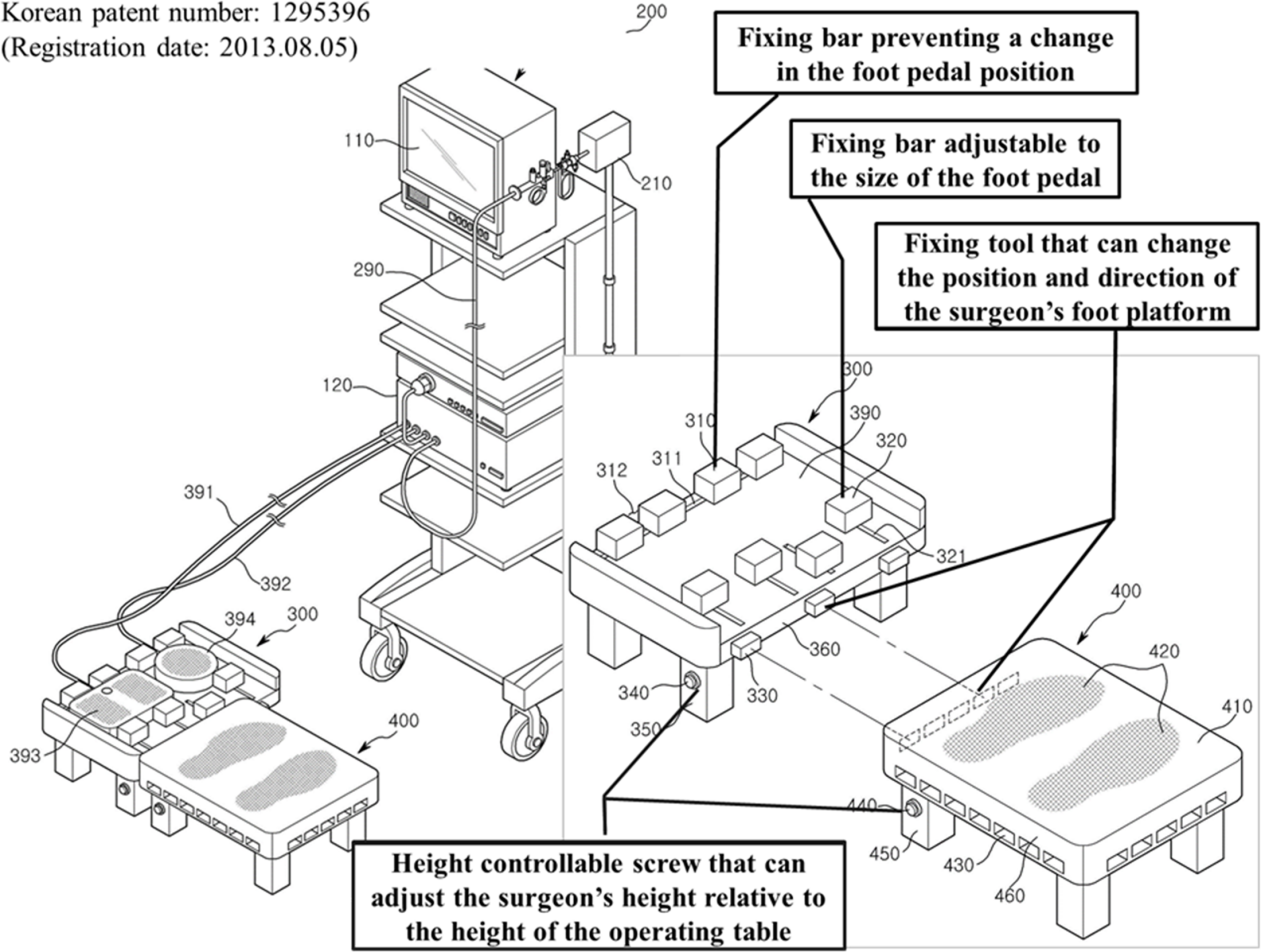
Schematic drawing of an ergonomic surgical step stool designed to reduce physical discomfort in laparoendoscopic surgeons.

Taking into account these study results, we designed an adjustable-height ergonomic surgical step stool for LES (Korean patent number 1295396; registration date: 2013.08.05; Fig 3). We think that controlling stool height can subsequently adjust the relative height of the monitor and operating table. Height-adjustable operating table can be more comfortable than our suggested foot stool, although this suggested foot stool can adjust the height of each LES in surgical team freely using the screw which can control the height of the stool. Our suggested foot stool was designed to adjust the height of LES freely using the screw which can control the height of the foot stool. This stool also has the possibility of offering greater foot and leg comfort by preventing the slippage of the foot pedal with the use of a wider adjustable plate combined with a fixing aid. It can fix the foot pedal regardless of the size of various foot switches using fixing tools, therefore, the LES do not need to spend efforts to find out the foot pedals when they wants to activate the foot switch. When we consider the previous report of 75% of the surgeons occasionally push the wrong switch [18], a dangerous situation for the patients, this stool may be not only comfortable but also safe. However, we cannot conclude about the effectiveness of this stool on the ergonomics and safety. A clinical study evaluating the ergonomic effectiveness of this step stool should be followed with a mock-up stool.

The strength of our study is the higher response rate of 52.4% compared with the response rate of 22% in the previous report with the largest sample survey found in the literature [10].

Moreover, the respondents were relatively homogeneous group, Korean LEGS with in the same specialty compared with the heterogeneous not only in the specialty but also in the nationality, in the previous reports [2,10,18].

Our research also had several limitations. First, each surgeon had a different duration of performing laparoscopic surgery; however, the loss of generalizability was slight because 67.6% of respondents were high-output LEGS. Second, the analysis was based on a questionnaire and did not include objective measures such as EMG. However, this questionnaire survey had a relatively high response rate in high-output LES performing with relatively homogeneous equipment in the same specialty and country. However we did not introduced any objective measurements of individuals, therefore numerous confounding variables exists that need to be corrected for more conclusive results.

In conclusion, we identified several factors associated with physical pain in LEGS. Knowledge of these factors will be useful to guide the implementation of ergonomic guidelines to relieve surgeons’ pain during LES. To overcome the anti-ergonomic conditions of the operating room, we proposed an ergonomic surgical step stool, the effectiveness of which should be evaluated in future studies.

## Supporting information

S1 File The response to the ergonomic questionnaires among laparoendoscopic gynecological surgeons. (XLS)
